# *Capsosiphon fulvescens* Glycoproteins Enhance Probiotics-Induced Cognitive Improvement in Aged Rats

**DOI:** 10.3390/nu12030837

**Published:** 2020-03-20

**Authors:** Jeong Hwan Oh, Taek-Jeong Nam, Youn Hee Choi

**Affiliations:** 1 Institute of Fisheries Sciences, Pukyong National University, Busan 46041, Korea; ojhwan55@pknu.ac.kr (J.H.O.); namtj@pknu.ac.kr (T.-J.N.); 2 Department of Marine Bio-Materials & Aquaculture, Pukyong National University, Busan 48513, Korea

**Keywords:** psychobiotics, aging-induced cognitive dysfunction, *Capsosiphon fulvescens*

## Abstract

Aging-induced cognitive dysfunction can be regulated by probiotics through bidirectional communication with the brain. This study aimed to investigate whether *Capsosiphon fulvescens* glycoproteins (Cf-hGP) enhanced probiotic-induced improvement of memory in aged rats and the underlying mechanism in the dorsal hippocampus. Cf-hGP were isolated using lectin resin. Cf-hGP (15 mg/kg/day) and/or *Lactobacillus plantarum* (*L. plantarum*) (10^9^ CFU/rat/day) were orally administered once a day for 4 weeks. Co-treatment with Cf-hGP and *L. plantarum* synergistically improved spatial memory in aged rats, which was overturned by functional blocks of brain-derived neurotrophic factor (BDNF) signaling. Increases in BDNF expression and nuclear factor erythroid 2-related factor 2 (Nrf2) phosphorylation were accompanied by mono- and/or co-administration in the dorsal hippocampus, while c-Jun N-terminal kinase (JNK) phosphorylation and glucose-regulated protein 78 expression were decreased. These synergistic effects were downregulated by blocks of BDNF/Nrf2-mediated signaling. In particular, co-treatment, not mono-treatment, reduced phosphorylation of eukaryotic elongation factor 2 (eEF2) regulated by eEF2 kinase and protein phosphatase 2A. Additionally, co-treatment downregulated the interaction between eEF2 kinase and JNK. These data demonstrated that cognitive impairment in aged rats was synergistically diminished by co-treatment with Cf-hGP and *L. plantarum* through BDNF-mediated regulation of Nrf2 and eEF2 signaling pathways in the dorsal hippocampus.

## 1. Introduction

Aging is a natural process associated with functional and cognitive decline, which is closely associated with neurobiological changes in the hippocampus, including a decrease in the number of newborn neurons in the subgranular zone of the hippocampus [1,2,3]. Further, volume reduction and vessel degeneration in the aged hippocampus may also contribute to reduced neurogenesis resulting in cognitive dysfunction [4,5]. Age-related neuroplasticity is closely linked to the microbiota-gut-brain axis, as well as to the proteostasis network [6].

Probiotics have been proposed as a key player in mental health. They are live microorganisms that confer a health benefit on the host when administered in adequate amounts [7]. The microbiota-gut-brain axis regulates behavioral and physiological abnormalities associated with neuroplasticity. Recently, many studies have shown that the gut-brain neural circuits affected by probiotics contribute to cognitive function, emotional disorders, such as anxiety and depression, and neurodegenerative diseases through direct and indirect pathways [8,9,10,11]. Moreover, these effects of probiotics on the nervous system are associated with neurotrophic factors [12,13].

Brain-derived neurotrophic factor (BDNF) plays an essential role in neuronal survival and growth, differentiation of new neurons, and hippocampal-dependent neuronal plasticity and long-term potentiation [14,15,16]. Aging is characterized by a decrease in BDNF concentrations, and chronic BDNF deficiency in the hippocampus induces cognitive dysfunction, in which BDNF dysregulation has been implicated in aging-associated psychiatric disorders [17,18,19,20]. In addition, BDNF-mediated changes in translation are necessary for long-lasting synaptic plasticity and memory, which is regulated by eukaryotic elongation factor 2 (eEF2) that is required for the translocation step in protein synthesis [21]. These data suggest that probiotic-induced retrieval of BDNF signaling in the hippocampus could reduce cognitive impairment in aged rats.

Neuronal proteostasis is essential for controlling synaptic plasticity and memory formation by regulating protein turnover in the ER, which plays a pivotal role in the correct synthesis, folding, and modification of proteins [22]. Aging-induced protein damage and alteration of the redox status can cause a decrease in the folding capacity and accumulation of misfolded proteins in the ER lumen to activate a series of signaling pathways, known as the endoplasmic reticulum (ER) stress response [23]. Further, alteration in proteostasis has been associated with aging-induced cognitive dysfunction and neurodegenerative diseases [24,25,26]. Glucose-regulated protein 78 (GRP78), a member of the heat shock protein family localized in the ER lumen, is a marker of ER stress [27]. Upon ER stress, GRP78 binds to unfolded proteins and activates a multi-chaperone complex, increasing the ER’s protein folding capacity [22]. The expression of GRP78 in hippocampus is associated with activation of c-Jun N-terminal kinase (JNK) signaling that plays critical roles in neuronal excitotoxicity and formation of amyloid plaques [28,29]. In addition, aging-induced alteration of redox homeostasis causes deregulation of nuclear factor erythroid 2-related factor 2 (Nrf2) that plays a crucial role in neurological disorders by regulating gene expression involved in antioxidant defenses [30]. Although various mechanisms regulate neuronal proteostasis, these data suggested that excessive ER stress with age can lead to dysregulation of neuronal proteostasis, consequently promoting cognitive disorders, and the BDNF-mediated regulation of ER stress may be closely associated with age-related hippocampal dysfunction and thus cognitive impairment.

An edible green alga, *Capsosiphon fulvescens* (*C. fulvescens*), is widely consumed as a nutrient source or functional food due to its beneficial health effects [31]. Extracts of *C. fulvescens* contain various antioxidants, such as polysaccharides and proteins [32,33,34,35]. Recent studies have demonstrated that crude proteins from *C. fulvescens* contribute to the enhancement of spatial memory in young and aged rats through activation of extracellular signal-regulated kinase 1/2 signaling by BDNF in a rat’s dorsal hippocampus [36,37]. However, the psychiatric effects of *C. fulvescens* remain unclear, as only a few studies related to this topic have been conducted.

Therefore, this study aimed to extend our current knowledge about the beneficial effects of *C. fulvescens* on psychological well-being against aging by investigating different aspects of probiotics. Thus, we investigated whether the hydrophilic compartments of *C. fulvescens* glycoproteins (Cf-hGP) enhanced the psychobiotic effect of *Lactobacillus plantarum* (*L. plantarum*) and its association with Cf-hGP-induced BDNF signaling in the dorsal hippocampus of aged rats.

## 2. Materials and Methods

### 2.1. Preparation of L. plantarum

*L. plantarum* is one of the most commonly administered probiotic strain, and thus the synergistic effect of Cf-hGP was assessed using type strain KCTC 3108 of *L. plantarum* which was distributed from the Korean Collection for Type Cultures (Jeongeup-si, Korea). Soon after, the strain was cultured at 37 °C for 18 h in the De Man, Rogosa, Sharpe broth (MRS; BD Difco, Becton-Dickinson, Sparks, MD, USA) and harvested by centrifugation at 6000 *g* for 10 min. The pellet was then resuspended in the MRS plus 25% glycerol to a final concentration of 2 × 10^9^ colony-forming units (CFU) per milliliter, which was aliquoted and stored at −80 °C until use. Before administration to rats, the aliquot was pre-warmed to 37 °C for 1 h and centrifuged at 6000 *g* for 10 min. The supernatants were discarded and resuspended in saline (0.9% NaCl) to give a concentration of 10^9^ per mL.

### 2.2. Isolation of Cf-hGP

To isolate the Cf-hGP enriched in the membrane, the wheat germ agglutinin lectin resin (Thermo Fisher Scientific, Rockford, IL, USA) was used that preferentially binds N-acetyl glucosamine commonly present in membrane glycoproteins. *C. fulvescens* was obtained from a farm on Wando island in Korea. Soon after, *C. fulvescens* powder (20 g) was mixed with 1 L of sodium acetate (pH 6.0), stirred at 4 °C overnight, and centrifuged at 5000 rpm for 10 min at 4 °C. The supernatant was transferred to a new 250 mL bottle and mixed at a ratio of 1:4:1:3 of the supernatant, methanol, chloroform, and distilled water, and the mixture was centrifuged at 12,000 rpm for 15 min at 4 °C. The supernatant above the interface was discarded. The remaining mixture was washed by adding three volumes of methanol and then centrifuging at 12,000 rpm for 15 min at 4 °C. The pellets were washed with methanol, resuspended in distilled water, transferred to a 50 mL Corning tube, and freeze dried overnight. To isolate hydrophilic glycoproteins for oral administration, the freeze dried powder was rehydrated with saline (0.9 % NaCl) and centrifuged at 13,000 rpm for 30 min at 4 °C. The supernatants were purified using the wheat germ agglutinin lectin resin (Thermo Fisher Scientific, MA, USA).

### 2.3. Scheme for Oral Administration

**Figure 1:**
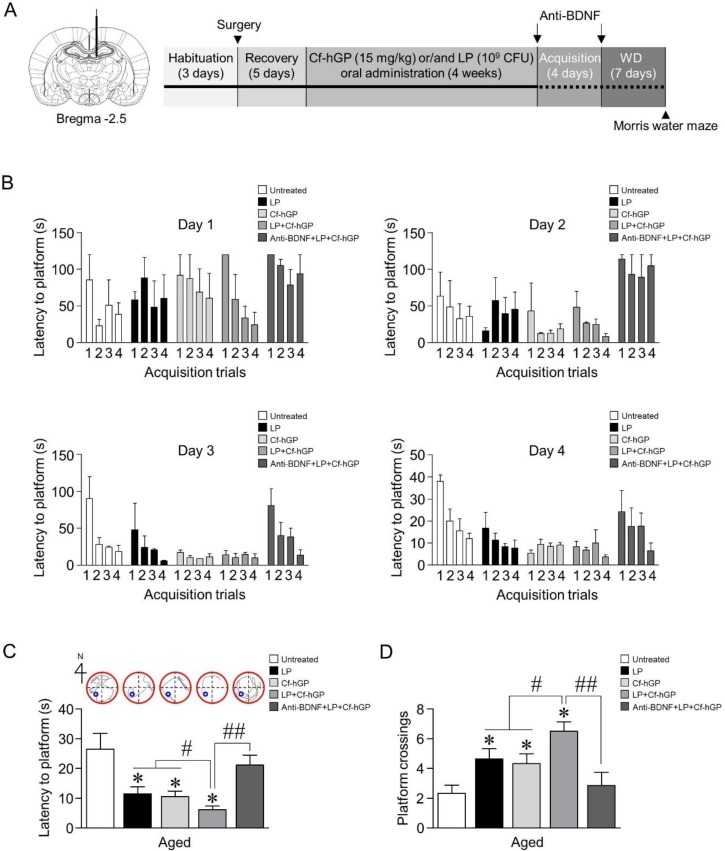
Effects of Cf-hGP on *L. plantarum*-induced cognitive improvement in aged rats. Cf-hGP (15 mg/kg) or *L. plantarum* (10^9^ colony-forming units (CFU)/rat/day) was orally administered once a day for 4 weeks (**A**). After four weeks, acquisition training was performed (**B**). The co-treatment with Cf-hGP and *L. plantarum* synergistically downregulated the latency and upregulated the frequency of platform crossings compared to mono-treatment (**C**,**D**). * *p* < 0.05 versus untreated rats; ^#^ *p* < 0.05 versus the rats treated with only Cf-hGP or LP; ^##^ *p* < 0.05 versus the rats co-treated with Cf-hGP and LP. Cf-hGP, *Capsosiphon fulvescens* glycoprotein; LP, *Lactobacillus plantarum*; BDNF, brain-derived neurotrophic factor; WD, withdrawal period.

Male Sprague Dawley rats (aged 12 months, 650–750 g; adolescent, 6 weeks, 150–180 g; *n* = 4–7 per each group) were obtained from Samtako Inc. (Gyeonggi, Korea). The rats were housed in pairs in a controlled environment and maintained on a 12-h light/dark cycle. Food and water were provided ad libitum. All animal experiments were approved by the Animal Ethics Committee of the Pukyong National University (PKNUIACUC-2019–23) and carried out in accordance with the guidelines for the care and use of laboratory animals. Oral administration schemes for Cf-hGP and/or *L. plantarum* are illustrated in Figure 1A. Soon after, the doses of Cf-hGP (15 mg/kg/day) and/or *L. plantarum* (10^9^ CFU/rat/day) or saline were determined based on the previous studies and were orally administered once a day for 4 weeks (Figure 1A) [10,37]. Following the last administration, the rats were subjected to acquisition training for four days with four trials per day. Seven days after the withdrawal, spatial learning memory was assessed based on escape latency.

### 2.4. Preparation of Crude Synaptosomal Fraction

The dorsal hippocampus was removed after the aged or adolescent rats were deeply anesthetized with a mixture of Zoletil 50 (18.75 mg/kg; Virbac Korea, Seoul, Korea) and Rompun (5.83 mg/kg; Bayer Korea, Seoul, Korea). Sections were serially cut using a microtome, and the dorsal hippocampus was removed using a steel borer (inner diameter of 2 mm). Lysis of hippocampal tissue samples was performed using a cold lysis buffer (pH 7.5) containing 0.32 M sucrose, 5 mM HEPES, and a protease inhibitor cocktail (Thermo Fisher Scientific). After centrifugation at 1000 *g* for 10 min at 4 °C to separate nuclei, the supernatant was transferred to a new 1.5 mL tube and centrifuged at 12,000 *g* for 10 min at 4 °C. After that, the supernatant was removed, and the pellet containing the crude synaptosome was used in this study [38].

### 2.5. Western Blot and Co-Immunoprecipitation

Protein concentrations were determined using a bicinchoninic acid protein assay kit (Thermo Fisher Scientific), and synaptosomal proteins (20 µg) were separated by 12% SDS-PAGE. Separated proteins were transferred to a polyvinylidene difluoride (PVDF) membrane. The membrane was blocked with a blocking buffer containing Tris-buffered saline (TBS), 1% bovine serum albumin, and 0.1% Tween 20, and then probed with primary antibodies for BDNF (Abcam, Cambridge, UK), Nrf2, phosphorylated (p)-Nrf2, GRP78, JNK, p-JNK, eEF2, p-eEF2, eEF2 kinase (eEF2K), p-eEF2K, and β-tubulin (Cell Signaling Technology, Danvers, MA, USA) at a dilution of 1:1000 each overnight at 4 °C on a shaker. After washing three times with TBS containing 0.1% Tween 20 for 10 min, membranes were incubated with a corresponding secondary antibody (Thermo Fisher Scientific) at a dilution of 1:10,000 for 60 min at room temperature. The membrane was stripped and reprobed with anti-β-tubulin antibody to normalize the blots. The chemiluminescent signals were detected by SynGene imaging system (Synoptics, Cambridge, UK) and were analyzed using the NIH ImageJ 1.48v software.

For co-immunoprecipitation (Co-IP), lysis of hippocampal tissues was performed using an IP lysis buffer containing 25 mM Tris-HCl (pH 7.4), 150 mM NaCl, 1% NP–40, 1 Mm ethylenediaminetetraacetic acid, 5% glycerol, and protease/phosphatase inhibitor cocktail (Thermo Fisher Scientific). After centrifugation at 13,000 *g* for 10 min at 4 °C, the supernatant was transferred to a new 1.5 mL tube for protein concentration. Proteins (500 µg) were combined with the eEF2K antibody in the manufacturer-recommended ratio of 1:50 and incubated overnight at 4 °C with mixing. Pre-washed Protein A/G Magnetic Beads (Thermo Fisher Scientific) were added and collected using a magnetic stand. After washing the beads, an SDS-PAGE sample buffer was added, and the supernatant was separated by 12% SDS-PAGE and transferred to a PVDF membrane. The membrane was blocked with a blocking buffer containing TBS, 1% bovine serum albumin, and 0.1% Tween 20, and then probed with primary antibodies for p-JNK and p-eEF2K (Cell Signaling Technology) at a dilution of 1:1000 each overnight at 4 °C on a shaker. Co-IP assay was performed repeatedly three times.

### 2.6. Double Immunofluorescence Staining

Double immunostaining was performed to confirm GRP78 expression in the rat dorsal hippocampus. Rats were deeply anesthetized with a mixture of Zoletil 50 (18.75 mg/kg; Virbac Korea, Seoul, Korea) and Rompun (5.83 mg/kg; Bayer Korea, Seoul, Korea), and then perfused with 4% paraformaldehyde in phosphate-buffered saline (PBS) at 4 °C. The brains were removed and post-fixed in a solution of 10% sucrose in 4% paraformaldehyde for 2 h at 4 °C, after which they were placed in 20% sucrose in PBS at 4 °C overnight. The perfused brains were serially cut in 30 μm sections using a sliding microtome on a freezing plate. Three sections per brain were used for immunohistochemistry. Following two washes with Dulbecco’s PBS (DPBS) with Ca^2+^ and Mg^2+^, brain sections (30 µm thickness) were fixed with 4% paraformaldehyde for 20 min and permeabilized with 0.3% Triton X-100 (diluted in DPBS with Ca^2+^ and Mg^2+^) for 5 min at room temperature. After three washes with PBS, the sections were incubated for 60 min at room temperature with a 5% goat serum solution in DPBS with Ca^2+^ and Mg^2+^ to block unspecific binding of the antibodies followed by incubation overnight at 4 °C in a mixture of rabbit anti-GRP78 and mouse anti-neuronal nuclear antigen (NeuN) primary antibodies (dilution 1:500). After washing three times with DPBS with Ca^2+^ and Mg^2+^, the cells were incubated in the mixture of two secondary antibodies (goat anti-rabbit IgG, Alexa Fluor 488, and goat anti-mouse IgG, Alexa Fluor 647) at a dilution of 1:500 for 60 min at room temperature. Following three washes and staining with the 4′,6-diamidino-2-phenylindole solution for 10 min, the cells were mounted with a drop of the ProLong gold antifade reagent (Gibco, Grand Island, NY, USA). Experiments were performed in triplicate tissue sections from each group (*n* = 4). Antibodies and normal goat serum for double immunostaining were purchased from Abcam. The fluorescent images were taken using an EVOS^®^ FL imaging system (Thermo Fisher Scientific). Quantification of GRP78 immunoreactivity was calculated using the ratio of intensities of GRP78 and NeuN-stained cells.

### 2.7. Rat Surgery and Microinjection

The aged or adolescent rats were deeply anesthetized with a mixture of Zoletil 50 (18.75 mg/kg; Virbac Korea, Seoul, Korea) and Rompun (5.83 mg/kg; Bayer Korea, Seoul, Korea). Under aseptic conditions, stainless steel guide cannulas were placed unilaterally to minimize surgical stress as described previously [37]. An infusion guide cannula (22 gauge; Plastics One, Roanoke, VA, USA) was implanted using the following coordinates from bregma: anterior-posterior, −2.5 mm; dorsal-ventral, −4.5 mm; medial-lateral, 1 mm. After a 28 gauge dummy cannula was inserted to prevent the guide cannula from clogging, the rats were allowed 5 days to recover. For postoperative care, rats were housed individually in a clean and quiet environment until they were fully ambulatory and daily monitored to see whether they ate and drunk. On the experiment day, following replacement with a 28 gauge internal cannula that protruded 0.5 mm beyond the guide cannula, function-blocking anti-BDNF antibody (1 µg/µL; Abcam), recombinant BDNF (1.5 µg/µL; R&D system, Minneapolis, MN, USA), or the Nrf2 inhibitor (ML385, 100 pmol/µL) was infused into the dorsal hippocampus, and the protein phosphatase 2A (PP2A) inhibitor (LB-100, 1.5 mg/kg) was administered by intraperitoneal injection. All drugs were purchased from Tocris Bioscience (Minneapolis, MN, USA) and diluted with an artificial cerebrospinal fluid. Further, the solutions were infused in a volume of 0.5 μL at a rate of 0.2 μL/min using a 2 µL Hamilton microsyringe (Reno, NV, USA) in freely moving rats at 30 min prior to the last oral administration of Cf-hGP and/or immediately after the acquisition training. After the microinjection, the internal cannula was left in place for an additional 5 min to prevent any possible backflow.

### 2.8. Morris Water Maze Test for Spatial Learning and Memory

Following the oral administration of Cf-hGP for 4 weeks, hippocampal-dependent learning and memory were assessed by the Morris water maze test. Soon after, using a stainless steel tank (diameter of 120 cm and depth of 45 cm), the platform was submerged 1 cm below the surface, and the water temperature was maintained at 25 °C. A set of semi-random starting positions was selected for basic acquisition training with the platform located in the southwest quadrant. Learning trials were conducted for four days (4 trials/day). Each trial was limited to 2 min, and the interval between the trials was 15 s. Seven days after the last learning trial, the reference memory was measured and assessed using the SMART 3.0 Video Tracking software (Harvard Apparatus, MA, USA).

### 2.9. Statistical Analysis

Significant differences in the number of immunoreactive pixels per measured area and spatial memory (the escape latency and the number of crossing platforms) were determined by the unpaired *t*-test or the one-way analysis of variance with repeated measures followed by the Tukey’s post-hoc test using GraphPad Prism 5 (GraphPad Software, La Jolla, CA, USA). Data are expressed as mean and the standard error of the mean for each group. The *p* value of ˂ 0.05 was considered significant.

## 3. Results

### 3.1. Co-Treatment with Cf-hGP and L. plantarum Synergistically Improves Spatial Memory in Aged Rats

We first investigated whether Cf-hGP administration enhanced probiotic-induced cognitive function in aged rats. Following oral administration of Cf-hGP (15 mg/kg) and/or *L. plantarum* (10^9^ CFU/rat/day) once a day for 4 weeks, spatial memory was measured using the Morris water maze (Figure 1A). After 4 weeks, acquisition training was performed. On day 4 of acquisition training, the latency to reach the platform decreased down to 30 s or less (Figure 1B). Further, seven days after the withdrawal, reference memory was assessed. The latency to reach the platform was significantly decreased [*F_(4,30)_* = 6.167; *p* < 0.05; *n* = 7 rats per group], while the frequency of platform crossings was increased [*F_(4,30)_* = 16.01; *p* < 0.05; *n* = 7 rats per group], for both treatment with only Cf-hGP and *L. plantarum* and co-treatment (Figure 1C,D). Interestingly, the co-treatment with Cf-hGP and *L. plantarum* synergistically downregulated the latency and upregulated the frequency of platform crossings compared to the mono-treatment. Since chronic Cf-hGP administration activates BDNF signaling in the dorsal hippocampus and improves spatial memory in aged rats [37], we further investigated whether the synergistic effects of co-treatment on spatial memory were associated with activation of BDNF signaling in the dorsal hippocampus. The anti-BDNF antibody (1 µg/µL) was unilaterally injected in the dorsal hippocampus (anterior-posterior, −2.5 mm; dorsal-ventral, −4.5 mm; medial-lateral, 1 mm from bregma) 30 min before the last administration and immediately after the last acquisition. As shown in Figure 1C,D, functional blocks of BDNF signaling with the anti-BDNF antibody overturned the synergistic effects caused by co-treatment with Cf-hGP and *L. plantarum*.

### 3.2. Synergistic Upregulation of Mature BDNF and Nrf2 Phosphorylation in the Dorsal Hippocampus by Cf-hGP Co-Treatment with L. plantarum is Accompanied by Downregulation of JNK Phosphorylation

**Figure 2:**
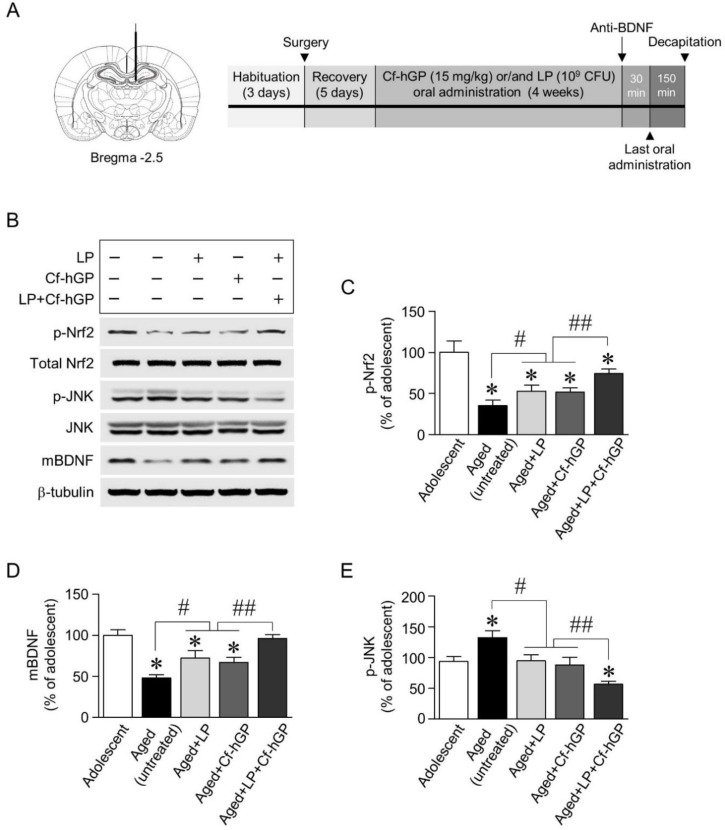
Expression of mature BDNF and phosphorylation of nuclear factor erythroid 2-related factor 2 (Nrf2) and c-Jun N-terminal kinase (JNK) in the dorsal hippocampus by co-treatment with Cf-hGP and *L. plantarum*. Following oral administration of Cf-hGP (15 mg/kg) or *L. plantarum* (10^9^ CFU/rat/day) once a day for 4 weeks, expression of mature BDNF (mBDNF) and phosphorylation of Nrf2 and JNK in the dorsal hippocampus were measured by immunoblotting (**A**,**B**). The co-treatment with Cf-hGP and *L. plantarum* synergistically increased Nrf2 phosphorylation and mBDNF expression compared to mono-treatment, but JNK phosphorylation was decreased (**C**–**E**). * *p* < 0.05 versus adolescent rats; ^#^ *p* < 0.05 versus aged untreated rats; ^##^ *p* < 0.05 versus the rats treated with only Cf-hGP or LP. Cf-hGP, *Capsosiphon fulvescens* glycoprotein; LP, *Lactobacillus plantarum*; p-Nrf2, phosphorylated Nrf2; p-JNK, phosphorylated JNK.

To investigate the underlying mechanisms for the synergistic effects of Cf-hGP on probiotic-induced enhancement in cognitive function, we explored whether chronic co-administration of Cf-hGP with *L. plantarum* regulated BDNF-mediated signaling linked to plasticity-related proteins, specifically, alterations of BDNF, Nrf2, eEF2, JNK, and GRP78, and their interactions in the dorsal hippocampus. We first examined expression of mature BDNF and phosphorylation of Nrf2 and JNK. Following oral administration of Cf-hGP (15 mg/kg) and/or *L. plantarum* (10^9^ CFU/rat/day) for 4 weeks (Figure 2A), protein expression was measured by immunoblotting. Mono-treatment with Cf-hGP or *L. plantarum* significantly upregulated phosphorylation of Nrf2 [*F_(4,20)_* = 32.00; *p* < 0.05; *n* = 5 rats per group] and expression of mature BDNF [*F_(4,20)_* = 25.51; *p* < 0.05; *n* = 5 rats per group] in the dorsal hippocampus, while JNK phosphorylation was downregulated compared to the aged untreated group [*F_(4,20)_* = 22.07; *p* < 0.05; *n* = 5 rats per group]. In particular, when Cf-hGP and *L. plantarum* were used for co-treatment for 4 weeks, Nrf2 phosphorylation and mature BDNF expression were synergistically increased, while JNK phosphorylation was decreased compared to mono-treatment (Figure 2B–E). There were no significant changes in the expression levels of JNK and Nrf2 in the dorsal hippocampus.

### 3.3. Blockade of BDNF Signaling Overturns Increases in Nrf2 Phosphorylation and Decreases in JNK Phosphorylation, While Inhibition of Nrf2 Signaling Downregulates Decreases in JNK Phosphorylation and ER Stress Response to Co-Treatment

**Figure 3:**
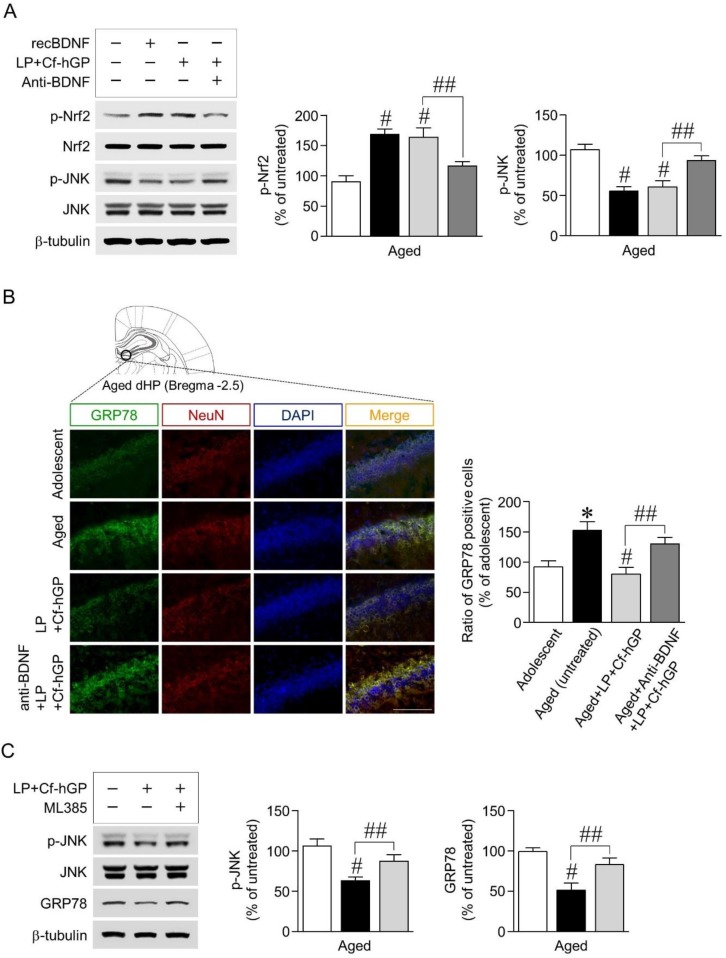
Effects of BDNF signaling activated by co-treatment with Cf-hGP and *L. plantarum* on Nrf2 and JNK phosphorylation and their interactions. The co-treatment-induced increase in Nrf2 phosphorylation was downregulated by functional blocks with the anti-BDNF antibody (1 µg/µL) (**A**). According to the double immunofluorescence staining, the co-treatment significantly downregulated the increase in glucose-regulated protein 78 expression in aged rats, which was overturned by blocks with the anti-BDNF antibody (1 µg/µL) (**B**). Inhibition of Nrf2 with ML385 (an inhibitor of Nrf2, 100 pmol/µL) overturned the co-treatment-induced decrease in JNK phosphorylation and GRP78 expression (**C**). * *p* < 0.05 versus adolescent rats; ^#^ *p* < 0.05 versus aged untreated rats; ^##^ *p* < 0.05 versus the rats co-treated with Cf-hGP and LP. Cf-hGP, *Capsosiphon fulvescens* glycoprotein; LP, *Lactobacillus plantarum*; recBDNF, recombinant BDNF; dHP, dorsal hippocampus; NeuN, neuronal nuclear antigen; DAPI, 4′,6-diamidino-2-phenylindole; GRP78, glucose-regulated protein 78. The scale bar represents 100 µm.

Next, we investigated whether BDNF signaling activated by co-treatment with Cf-hGP and *L. plantarum* contributed to phosphorylation of Nrf2 and JNK and their interactions in the dorsal hippocampus. Recombinant BDNF (1.5 µg/µL) as a positive control was unilaterally injected into the dorsal hippocampus, which significantly increased Nrf2 phosphorylation [*F_(3,20)_* = 35.43; *p* < 0.05; *n =* 6 rats per group], but JNK phosphorylation was decreased [*F_(3,20)_* = 46.15; *p* < 0.05; *n =* 6 rats per group]. The co-treatment-induced increase in Nrf2 phosphorylation was downregulated by functional blocks with the anti-BDNF antibody (1 µg/µL) (Figure 3A). We further investigated whether the co-treatment-induced BDNF signaling-regulated ER stress response was associated with JNK phosphorylation. Expression of GRP78, an ER stress marker, was significantly increased in aged rats (12 months) compared to adolescent rats (6 weeks) as assessed by double immunofluorescence staining. The co-treatment with Cf-hGP and *L. plantarum* significantly downregulated the increase in GRP78 expression in aged rats, which was overturned by blocking BDNF signaling with the anti-BDNF antibody (1 µg/µL) [*F_(3,12)_* = 12.46; *p* < 0.05; *n =* 4 rats per group] (Figure 3B). In addition, we determined that JNK phosphorylation and GRP78 expression were regulated by the activated BDNF-mediated Nrf2 signaling pathway, stimulated by the co-treatment. As shown in Figure 3C, inhibition of Nrf2 with ML385 (100 pmol/µL) overturned the co-treatment-induced decrease in JNK phosphorylation [*F_(2,15)_* = 27.85; *p* < 0.05; *n =* 6 rats per group] and GRP78 expression [*F_(2,15)_* = 32.90; *p* < 0.05; *n =* 6 rats per group] in the dorsal hippocampus.

### 3.4. Blockade of BDNF Signaling or Inhibition of PP2A Overturned Decrease in eEF2 Phosphorylation by Co-Treatment with Cf-hGP and L. plantarum

**Figure 4:**
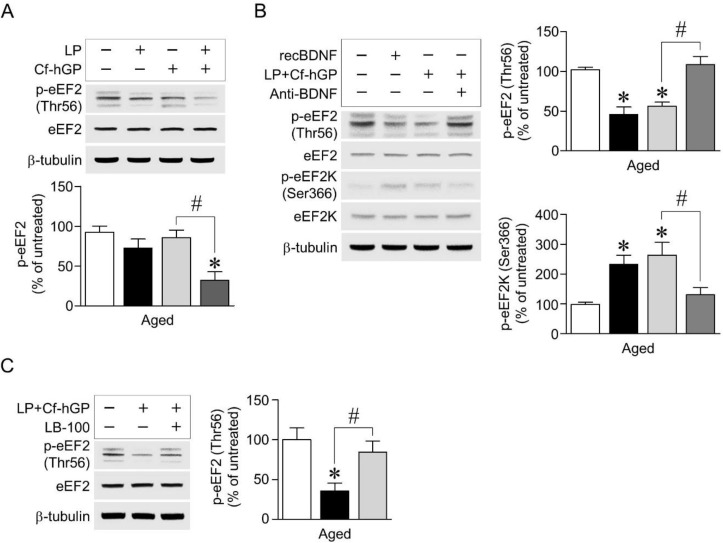
Regulation of BDNF-mediated phosphorylation of eukaryotic elongation factor 2 (eEF2) by co-treatment with Cf-hGP and *L. plantarum*. Phosphorylation of eEF2 at threonine 56 was decreased by co-treatment with Cf-hGP and *L. plantarum*, but not by any mono-treatment (**A**). Phosphorylation of the eEF2 kinase was upregulated, while eEF2 phosphorylation was downregulated by the co-treatment, which was overturned by functional blocks with the anti-BDNF antibody (1 µg/µL) (**B**). The co-treatment-induced decrease in eEF2 phosphorylation was downregulated by inhibition of protein phosphatase 2A (PP2A) with LB-100 (an inhibitor of PP2A, 1.5 mg/kg) (**C**). * *p* < 0.05 versus untreated rats; ^#^ *p* < 0.05 versus the rats co-treated with Cf-hGP and LP. Cf-hGP, *Capsosiphon fulvescens* glycoprotein; LP, *Lactobacillus plantarum*; eEF2K, eEF2 kinase; p-eEF2K, phosphorylated eEF2K; Thr56, threonine 56; Ser366, serine 366.

Since activation of BDNF-mediated Nrf2 signaling protects neurons against ER stress by regulating protein synthesis, including antioxidant proteins [39], we further investigated whether the co-treatment-induced BDNF signaling activated eEF2, which is a critical regulator in the translation step [40]. The co-treatment with Cf-hGP and *L. plantarum* downregulated eEF2 phosphorylation at threonine 56 [*F_(3,24)_* = 22.71; *p* < 0.05; *n =* 7 rats per group]; however, there were no significant differences in the mono-treatment with Cf-hGP or *L. plantarum* (Figure 4A). In addition, the co-treatment significantly upregulated eEF2K phosphorylation [*F_(3,20)_* = 42.67; *p* < 0.05; *n =* 6 rats per group], while it reduced eEF2 phosphorylation [*F_(3,20)_* = 20.89; *p* < 0.05; *n =* 6 rats per group], which was overturned by functional blocks with the anti-BDNF antibody (1 µg/µL) (Figure 4B). The co-treatment-induced decrease in eEF2 phosphorylation was also downregulated by inhibition of PP2A with LB-100 (1.5 mg/kg) [*F_(2,15)_* = 25.29; *p* < 0.05; *n =* 6 rats per group] (Figure 4C).

### 3.5. Co-Treatment with Cf-hGP and L. plantarum Reduces Interaction between eEF2K and JNK

**Figure 5:**
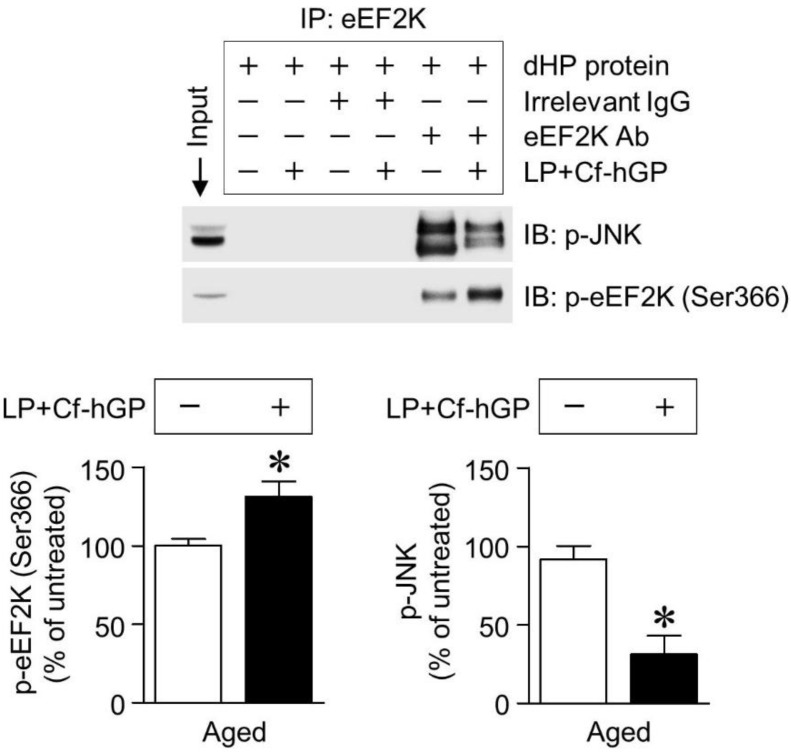
Effect of co-treatment with Cf-hGP and *L. plantarum* on the eEF2 kinase-JNK interaction. JNK was immunoprecipitated using the eEF2 kinase antibody. The interaction between the eEF2 kinase and JNK was downregulated by co-treatment with Cf-hGP and *L. plantarum*. Further, a significant decrease in JNK phosphorylation was accompanied by an increase in eEF2 kinase phosphorylation. * *p* < 0.05 versus untreated rats; unpaired *t*-test. Cf-hGP, *Capsosiphon fulvescens* glycoprotein; LP, *Lactobacillus plantarum*; eEF2K, eEF2 kinase; dHP, dorsal hippocampus; IP, immunoprecipitation; IgG, immunoglobulin G; Ab, antibody.

Finally, we investigated whether eEF2K interacted with JNK in the dorsal hippocampus, and the effect of co-treatment on this interaction. In co-immunoprecipitation with the eEF2K antibody using extracts from the dorsal hippocampus, co-treatment with Cf-hGP and *L. plantarum* significantly increased eEF2K phosphorylation (unpaired *t*-test, *t* = 2.260; *df* = 6; *p* < 0.05; *n =* 4 rats per group) and induced its inactivation, which downregulated the interaction between eEF2K and JNK and reduced JNK phosphorylation (unpaired *t*-test, *t* = 6.826; *df* = 6; *p* < 0.05; *n =* 4 rats per group) in the dorsal hippocampus (Figure 5).

## 4. Discussion

Brain aging causes cognitive impairment that negatively affects learning and memory, which are mainly associated with the dorsal hippocampus. In recent years, many studies have demonstrated that probiotics play a crucial role in cognitive function through bidirectional communication with the brain. Therefore, this study aimed to investigate whether chronic Cf-hGP administration enhanced the probiotic-induced improvement of memory in aged rats and its association with activation of BDNF-mediated Nrf2 and eEF2 signaling pathways in the dorsal hippocampus.

Probiotics play a crucial role in various physiological processes and majorly impact the cognitive function and emotional disorders. The effects of these probiotics on psychiatric diseases are associated with BDNF, which plays an essential role in neuronal survival and neurogenesis in dentate gyrus of the hippocampus, and in new synapse formation for learning and memory [41]. In this study, mono-treatment with Cf-hGP or *L. plantarum* for 4 weeks significantly downregulated the latency to platform and upregulated the frequency of platform crossings in the Morris water maze test (Figure 1C,D). Interestingly, the co-treatment with Cf-hGP and *L. plantarum* synergistically enhanced spatial memory in aged rats, which was overturned by blocking hippocampal BDNF signaling with the anti-BDNF antibody (1 µg/µL). Next, to explore these synergistic effects caused by Cf-hGP, we further determined that BDNF-mediated signaling was differentially regulated by the co-treatment in the dorsal hippocampus, which is a pivotal region in spatial memory [42]. As shown in Figure 2B, mature BDNF expression and Nrf2 phosphorylation were significantly increased by each mono-treatment, while JNK phosphorylation was downregulated. As shown by the behavior test, co-treatment with Cf-hGP and *L. plantarum* synergistically upregulated the alterations caused by mono-treatment (Figure 2C–E).

Since BDNF induces activation of signaling of transcription factor Nrf2, which is a key regulator of antioxidant protein expression against oxidative damage and ER stress [30], we further investigated whether the synergistic enhancement of BDNF signaling regulated Nrf2 and JNK phosphorylation, and whether activation of BDNF-mediated Nrf2 signaling was associated with the co-treatment-induced decrease in JNK phosphorylation and ER stress response. When the hippocampal BDNF signaling was activated by injection of recombinant BDNF (1.5 µg/µL) or the co-treatment, Nrf2 phosphorylation was significantly increased, while JNK phosphorylation and GRP78 expression were decreased; these changes were overturned by blocking BDNF signaling with the anti-BDNF antibody (1 µg/µL) (Figure 3A,B). In addition, inhibition of Nrf2 signaling with ML385 (100 pmol/µL) eliminated the co-treatment-induced decrease in JNK phosphorylation and GRP78 expression (Figure 3C). GRP78, a member of the heat shock protein family localized in the ER lumen, is a marker of ER stress [27]. Upon ER stress, GRP78 binds to unfolded proteins and activates a multi-chaperone complex, resulting in increased ER protein folding capacity [22]. ER stress with age induces dysregulation of proteostasis, which can modulate synaptic activity; thus, ER stress could be one of the major risk factors for cognitive dysfunction [26,43]. Collectively, these findings suggested that chronic administration of Cf-hGP with *L. plantarum* could positively increase psychobiotic effects of *L. plantarum* compared to the mono-treatment through activation of BDNF-mediated Nrf2 signaling in the dorsal hippocampus.

In addition, protein synthesis in neurons is tightly regulated for proteostasis and synaptic plasticity, which are closely associated with long-term maintenance of memory [44]. In particular, translational control of plasticity-related proteins is important for memory consolidation [45]. When additional amino acids are added to the growing peptide in the elongation step, elongation factor 2 (eEF2) mediates translocation of peptidyl-transfer ribonucleic acid from the ribosomal A-site to the P-site [40]. In mammals, eEF2 is phosphorylated at threonine 56 (Thr56) by eEF2K, resulting in inactivation of eEF2 and inhibited elongation. Thus, regulation of eEF2 phosphorylation is necessary for neuronal plasticity to improve memory consolidation [21,46].

In this study, only the co-treatment with Cf-hGP and *L. plantarum* significantly reduced eEF2 phosphorylation (Thr56), not any mono-treatment (Figure 4A). The decrease in eEF2 phosphorylation (Thr56) was accompanied by an increase in eEF2K phosphorylation, which was downregulated by blocks with the anti-BDNF antibody (1 µg/µL) (Figure 4B). Further, the decreased Nrf2 phosphorylation was overturned by inhibition of PP2A with LB-100 (1.5 mg/kg) (Figure 4C). In addition, the co-treatment with Cf-hGP and *L. plantarum* significantly increased eEF2K phosphorylation and inactivated eEF2K, which reduced its interaction with JNK and JNK phosphorylation in the dorsal hippocampus (Figure 5B,C). These data demonstrated that the activation of hippocampal BDNF signaling by the co-treatment downregulated phosphorylation of eEF2 linked to the eEF2K and PP2A in the dorsal hippocampus, suggesting that regulation of eEF2 phosphorylation may be a key underlying mechanism of enhancing the synergistic effects of Cf-hGP on the cognitive function in aged rats.

## 5. Conclusions

**Figure 6:**
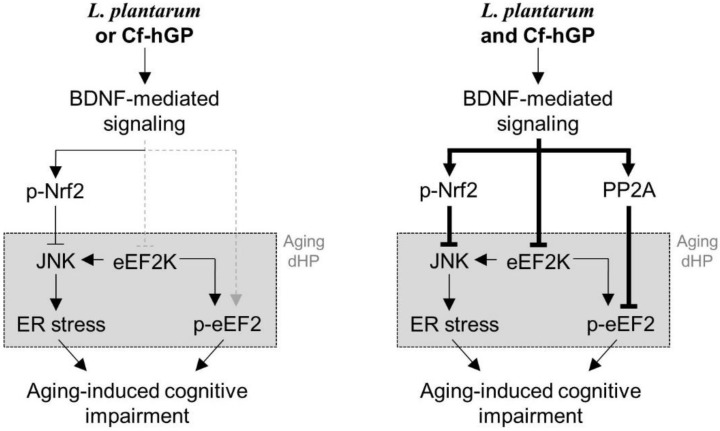
Scheme of a proposed mechanism of underlying effects of Cf-hGP on *L. plantarum*-induced cognitive improvement in aged rats. Chronic administration of Cf-hGP with *L. plantarum* synergistically attenuated cognitive impairment in aged rats through BDNF-mediated control of Nrf2 and eEF2 signaling in the dorsal hippocampus. Cf-hGP, *Capsosiphon fulvescens* glycoprotein; LP, *Lactobacillus plantarum*; dHP, dorsal hippocampus; ER, endoplasmic reticulum.

These data demonstrate that chronic co-administration of Cf-hGP with *L. plantarum* could synergistically improve the cognitive function in aged rats compared to any mono-treatment through activation of BDNF-mediated Nrf2 and eEF2 signaling in the dorsal hippocampus (Figure 6). Therefore, these findings provide new insights of the potential roles of seaweed glycoproteins in the gut-brain axis, which can be used to develop new applications for psychological well-being.
